# Open-source sensor for measuring oxygen partial pressures below 100 microbars

**DOI:** 10.1371/journal.pone.0206678

**Published:** 2018-11-14

**Authors:** Mihkel Pajusalu, Cauê S. Borlina, Sara Seager, Shuhei Ono, Tanja Bosak

**Affiliations:** Department of Earth, Atmospheric and Planetary Sciences, Massachusetts Institute of Technology, Cambridge, Massachusetts, United States of America; Istituto Italiano di Tecnologia Center for Micro BioRobotics, ITALY

## Abstract

The ability to measure partial pressures of oxygen below 100 microbars and nanomolar dissolved oxygen concentrations in *in situ* laboratory systems benefits many fields including microbiology, geobiology, oceanography, chemistry, and materials science. Here, we present an easily constructible open-source design for a networked luminescence lifetime measurement system for *in situ* measurements in arbitrary laboratory containers. The system is well suited for measuring oxygen partial pressures in the 0–100 μbar range, with the maximum potentially usable upper range limit at around 10 mbar, depending on experimental conditions. The sensor has a limited drift and its detectability limit for oxygen is at 0.02 μbar for short timescale measurements. Each sensor can connect to a Wi-Fi network and send the logged data either over the Internet or to a local server, enabling a large number of parallel unattended experiments. Designs are also provided for attaching the sensor to various commercially available containers used in laboratories. The design files are released under an open source license, which enables other laboratories to build, customize, and use these sensors.

## Introduction

The ability to precisely determine trace oxygen concentrations is required in many fields, including microbiology, geology, geobiology, oceanography, chemistry, and materials science. For example, to confirm anaerobic conditions similar to those inferred for the early Earth, and to measure oxygen concentration in natural anoxic zones *in situ* [1], capability of detecting parts per million mixing ratios of oxygen is required. In geology, low oxygen sensing can be used to quantify the rates at which minerals weather as a function of oxygen concentration [2]. More generally, oxygen concentration sensors are required to quantify oxygen diffusion into systems and verify the quality of preparation facilities such as anaerobic chambers.

Several sensors for measuring trace oxygen concentration already exist [3] and are employed in various measurement situations. In this manuscript, we will concentrate on sensors that can be used to measure trace oxygen sensors in user selectable small volume containers (mainly lab glassware) *in situ*. These sensors are generally based on detecting oxygen-induced changes in the luminescence lifetime of a dye, i.e. the time it takes for the luminescence signal to decay after excitation. By exciting the dye with a sine-wave modulated excitation and measuring the phase shift between excitation and emission, it is possible to measure the luminescence lifetime very precisely. Low oxygen concentrations can also be measured by electrochemical sensors.

Some examples of commercial sensors are Oxygen Sensor Spot and Trace Oxygen Sensor Spot from Pyro Science (Pyro Science GmbH, Aachen, Germany) [4]. When these two sensors are used with the proprietary readout systems, they can measure dissolved oxygen concentrations with 160 nM and 31 nM resolution, respectively, and with 310 nM and 62 nM limits of detection, respectively. RedEye FOSPOR [5] from OceanOptics (Largo, Florida, USA) has a 2.6 nM resolution. Its lower detection limit has not been specified by the manufacturer, it is expected to be at least 5.2 nM. The lower detection limits of these instruments may be too high to monitor some microaerophilic or nanoaerophilic metabolisms under conditions relevant for the suboxic areas of the modern and Archean oceans, where the dissolved oxygen concentrations are thought to have been 2.2 nM or lower [6]. An improvement upon these sensors are LUMOS [7–9] and STOX [1] sensors. STOX sensors can detect down to 2 nM of dissolved oxygen with the approximately 30 minute integration time [1] and LUMOS sensors have a detection limit of around 0.5 nM [7]. However, neither of these sensors is commercially available.

The main purpose of this report is to present an open-source sensor design and describe in detail the optimization of calibration procedures in the low-nanomolar range. Both the design and clear calibration procedures are required to increase the availability of precise sensors that can detect trace oxygen *in situ* in laboratory vessels. Additionally, large scale experiments rely on sensors that can communicate wirelessly and be autonomous for the duration of an experiment. We take the LUMOS sensor as a starting point and improve on the previous sensor designs by reducing noise, reducing and quantifying sensor drift, developing and describing the details of the calibration procedure, and connecting the sensors to a WiFi network. The release of these results into the open-source ecosystem will enable other laboratories to use the same sensors or modify the current design for their own needs. The same readout devices can be adapted to work with different dyes that sense different environmental conditions.

## Theory of operation

The sensor detects oxygen concentration by measuring the change in the luminescence lifetime of a luminescent dye. This method is used, among others, by the LUMOS sensor [7]. In simplest approximation, the decay of emission intensity *I*(t) can be approximated by an exponential decay function with a time constant *τ*, which is referred to as the luminescence lifetime:

$$I\left(t\right)=I_{\max}\exp\left(-t/\tau \right)$$ (1)

**Fig 1:**
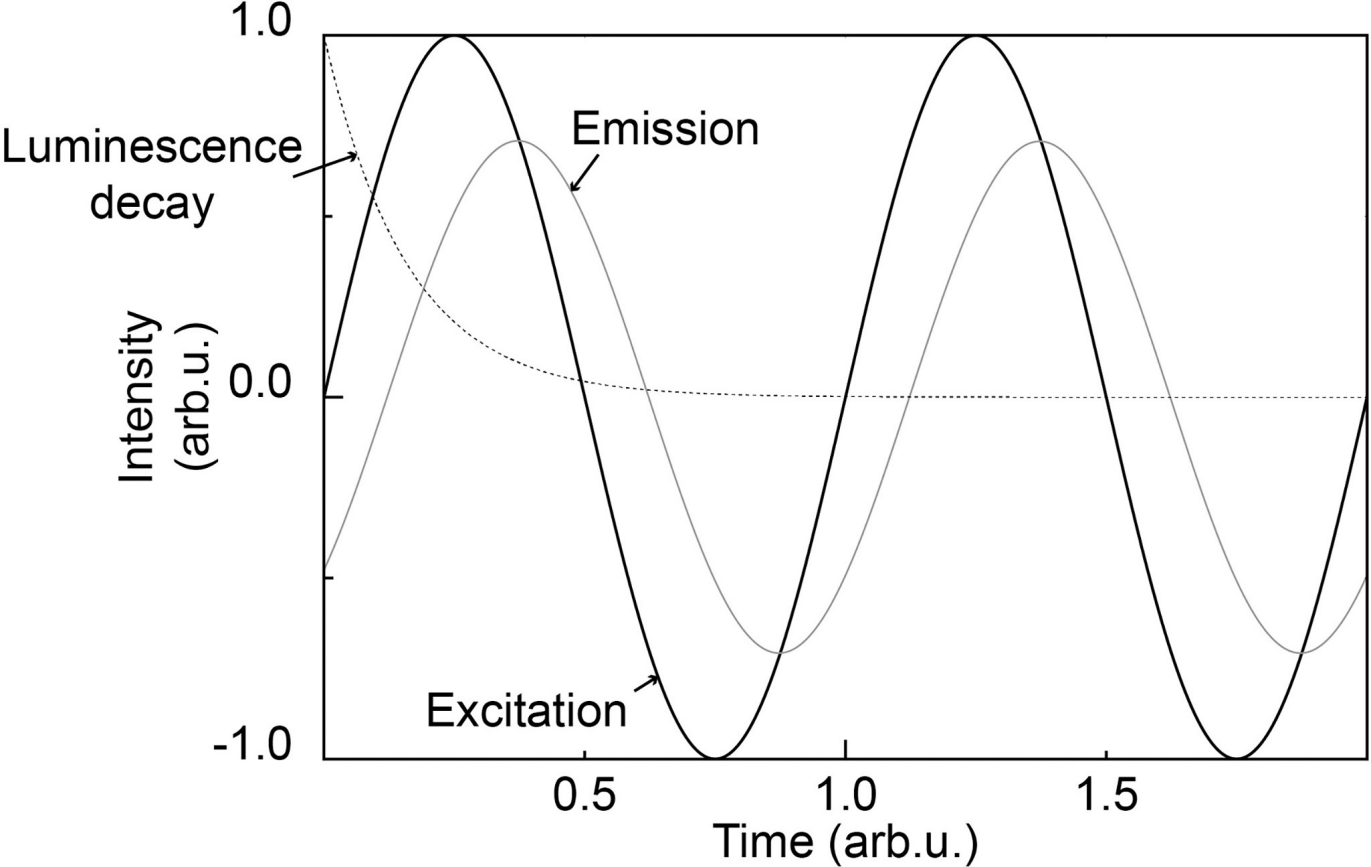
General operating principle of luminescence lifetime sensors based on sine wave attenuation.

If a dye is excited with a sine-wave modulated signal, the luminescence will also be a sine wave, but phase shifted (see Fig 1).

$$I\left(t\right)=I_{\max}\sin\left(2\pi tf-2\pi \tau f\right)$$ (2)

Measuring the phase shift is preferable, because it can be computationally efficiently extracted by using single frequency Fourier transform.

$$2\pi \tau f=-\frac{\int I\left(t\right)\sin(2\pi ft)dt}{\int I\left(t\right)\cos(2\pi ft)dt}$$ (3)

To optimize this process for the limited memory and processing resources of microcontrollers, the readings can be accumulated into a buffer that has the same length as one period of the excitation signal. The Fourier’ transform can then be performed on the buffer after the acquisition cycle. This is especially efficient because the buffer addition can be performed using integers, allowing faster sampling rates and the acquisition of more signal samples per sampling period, even on general-purpose microcontrollers.

$$I\left[t\right]=\sum_{k=0}^{cycles}I\left(t+k\cdot f\right)$$ (4)

In addition to computational efficiency, the summed waveform can be used to evaluate the signal quality visually by plotting the signal on a display attached to the system, making it easier to optimize measurement conditions.

**Fig 2:**
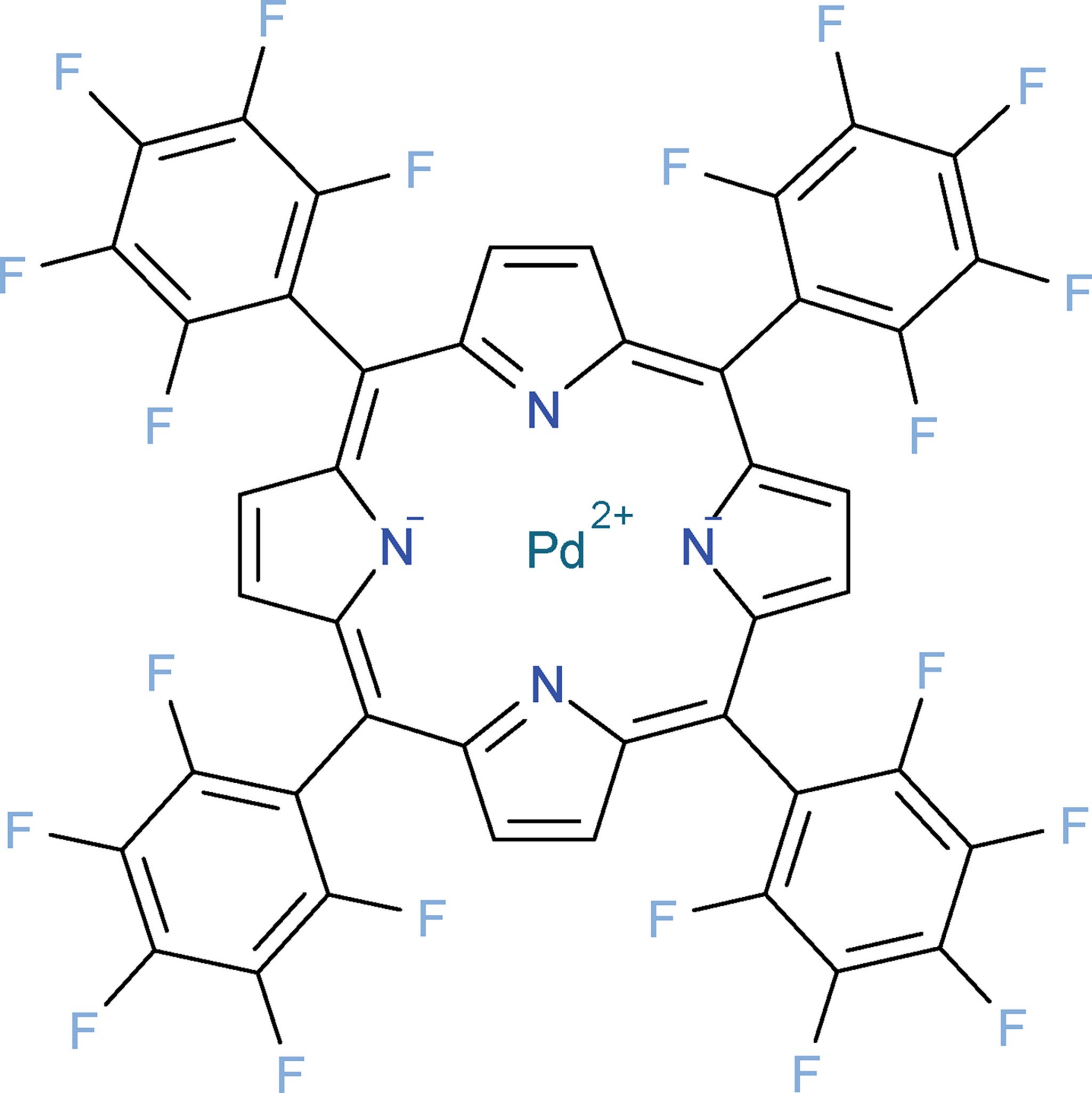
Chemical structure of 5,10,15,20-Tetrakis(pentafluorophenyl)-21H,23H-porphyrin palladium(II) [10], the dye used in our sensor patches. Image generated from PubChem data using OpenBabel **[12]**.

Our setup uses the luminescent dye palladium(II)-5,10,15,20-tetrakis-(2,3,4,5,6-pentafluorphenyl)-porphyrin [10], abbreviated Pd-TFPP (Fig 2), just as the LUMOS sensor [7]. The luminescence lifetime and yield of this dye depend on the partial pressure of oxygen and are negligible at atmospheric levels, but increase to easily measurable levels when oxygen is not present [7]. For convenience, we use microbars (0.1 Pa) as the measurement unit in the subsequent text to quantify oxygen abundance. According to Henry’s law, this partial pressure of oxygen, equilibrated with distilled water, produces, according to Henry’s law, a dissolved oxygen concentration of 1.3 nM at 25°C [11]. At the total pressure of 1 atmosphere this partial pressure is equivalent to the mixing ratio of 0.987 parts per million.

The sensor system modulates a light-emitting diode (LED) with a sinusoidal excitation waveform and records the emission waveform using a photodiode. Exciting beam and emitted radiation pass through filters to minimize the amount of exciting light that reaches the detector. In practice, however, this filtering cannot be done perfectly, and the recorded emission signal will contain the excitation signal [7]. A similar effect can also happen through the unwanted coupling of signals inside the electronics. Fourier transform is extremely selective towards the component with the selected frequency, but given that the leaked excitation signal has the same frequency as the emission, digital filtering alone cannot uncouple the signals. This can cause unwanted phase shifts in the measured waveform. To solve this issue, we add a step to measurement process: the photodiode signal is measured in the presence of the identical excitation, detector placement, temperature and other conditions as in the actual experiment, but with high oxygen partial pressure around the dye. Under these conditions, the luminescence signal will be negligible, and all the received signal can be attributed to either the couplings in electronics or the excitation signal that reaches the photodiode. This signal is independent of the partial pressure of oxygen, so it can be recorded and subtracted from the total measured signal.

## Sensor design

**Fig 3:**
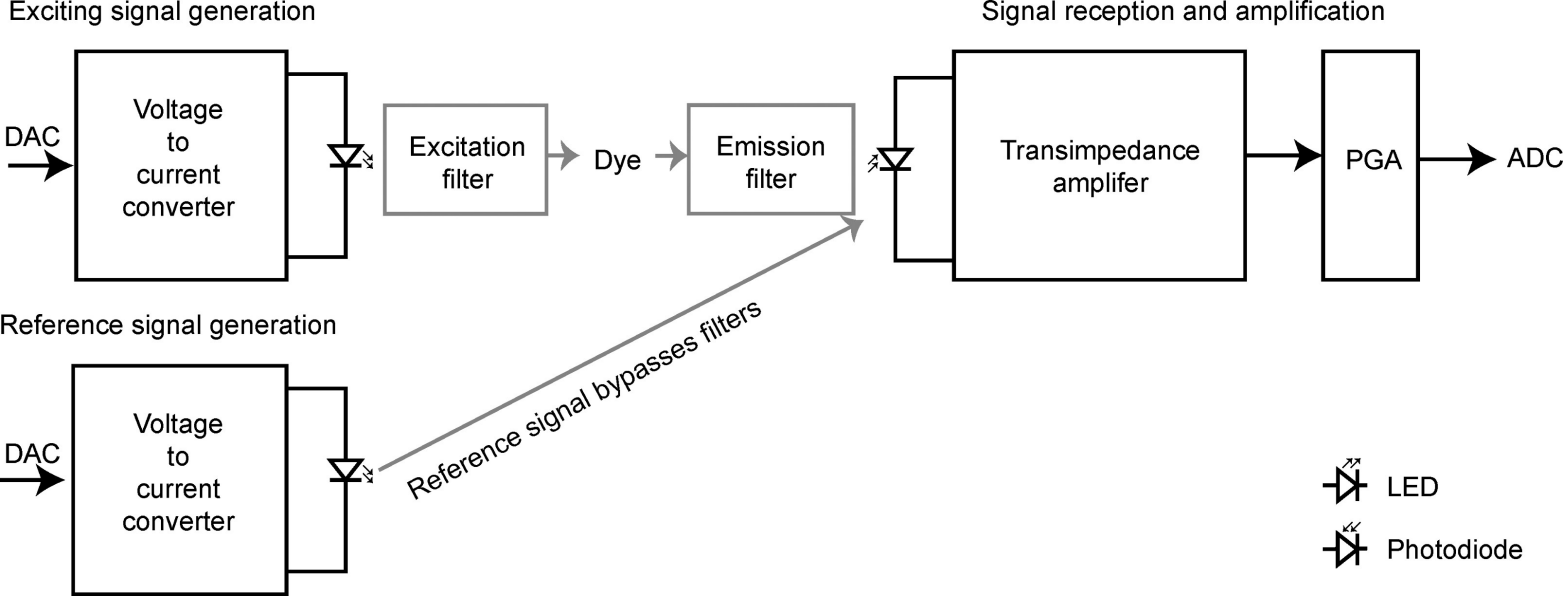
General operating principle of luminescence lifetime sensors based on the sine wave phase shift. DAC is the digital to analog converter, PGA is the programmable gain amplifier and ADC is the analog to digital converter.

The starting point for the development of this sensor was the published description of the LUMOS sensor [7], but the exact implementation was designed from ground up. The operational principle is shown in Fig 3. The optical dye is deposited on a glass disc. The coated disc is placed in the measurement container and a readout system is used to measure the luminescence lifetime. In our design, the sensor patch is kept in the headspace and it measures the partial pressure of oxygen in the gas phase. The concentrations of dissolved oxygen, if needed, can be calculated from Henry’s Law [11]. This geometry avoids direct contact between the sensor patch and bacterial cultures, and directs the exciting light away from the culture.

The readout system consists of two LEDs with their respective modulation circuits, and a photodiode with a transimpedance amplifier and a programmable gain amplifier (PGA). The microcontroller digital-to-analogue converter (DAC) generates the excitation signal and the final signal is recorded using the microcontroller analog to digital converters (ADC). The system also contains a second LED, the emission from which bypasses the filters and reaches the photodiode directly. The second LED can be used to measure the phase shift between the emitted and the received signals caused by phase delays in the electronics board itself. This functionality is provided for compatibility with the LUMOS sensor, although the applications presented in the current paper did not require its use.

To maximize the utility of the sensors in the open-source ecosystem, we designed the luminescence lifetime measurement system as an add-on module (also known as a shield) for the Arduino Due [13] open-source electronics prototyping board. This platform was selected because it has two 12-bit digital-to-analog converters and eight 12-bit analog-to-digital converters that can be operated at nearly 1 million samples per second, and a large number of general purpose input/output pins. The microcontroller on Arduino Due is a 32-bit Atmel SAM3X8E ARM Cortex-M3 microcontroller [14] that operates at 84 MHz and can perform all the required signal processing. The Arduino Due board also contains an internal temperature sensor, which can be used to correct for temperature-induced drifts.

**Fig 4:**
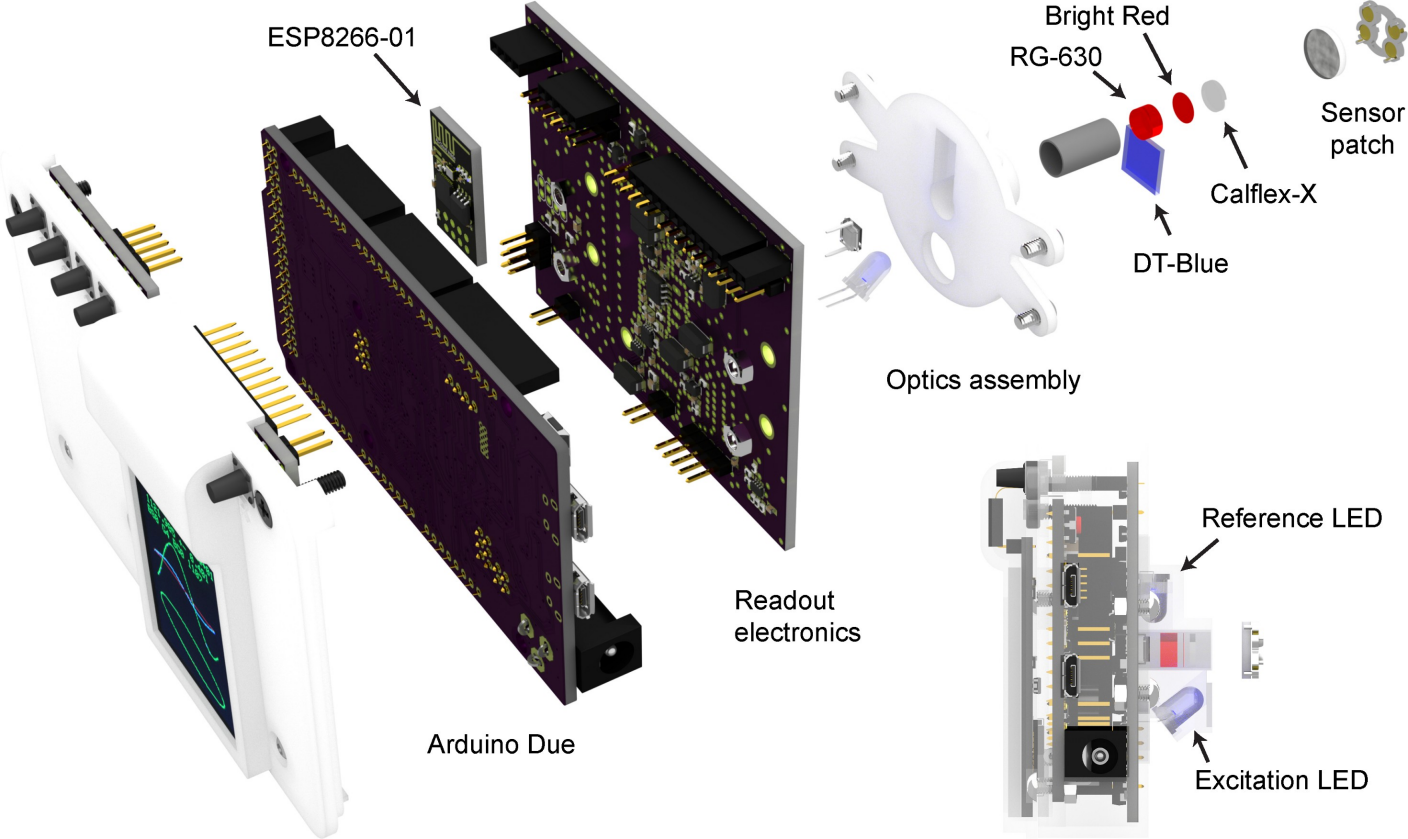
Schematic rendering of the system. Rendered in Blender 2.78 using Cycles rendering engine. Circuit board models are constructed by a heavily modified version of PCB to Blender **[16]** that uses redesigned component models. Front cover is not shown in the exploded drawing for clarity.

Fig 4 shows a computer rendering of the whole system and details the physical layout of mechanical, electrical, and optical components. The system is centered around the readout electronics board to which the photodiode and LEDs attach. The printed circuit board (PCB) also contains holes for mounting the 3D printed optics assembly holder that houses optical filters. The sensor board has connections to attach an LCD (liquid crystal display) screen with a micro SD (secure digital) card holder (1.8 inch TFT from SainSmart, sainsmart.com), a strip of buttons on a PCB for operation and a reset button PCB and an ESP8266-01 module [15] for communication over 2.4 GHz WiFi.

### Optical assembly

The individual components of the optics (see Fig 4) are almost identical to the ones in LUMOS sensor [7]. The emitted signal from the light diode (405 nm T-1 ¾ LED from Visual Communications Company, San Marco, California, USA) is filtered by a 10 mm x 10 mm highpass filter (DT-Blue from Qioptiq Photonics, Goettingen, Germany). The emitted signal is received through a Calflex-X IR-blocking filter (from Qioptiq Photonics, Goettingen, Germany), a #026 Bright Red film filter, and a RG-630 colored class filter (Edmund Optics, Barrington, New Jersey, USA). The photodiode used was BPW34 (Vishay Intertechnology, Malvern, Pennsylvania, United States). The receiving optics are housed in a 7.2 mm outer diameter 6.4 mm inner diameter aluminum tube and the entire assembly is fixed to a 3D printed nylon holder (see Fig 3).

All 3D printed nylon parts were produced by ShapeWays (New York City, New York, USA). The optical filters were cut into appropriate size by Ferro Ceramic Grinding (Wilmington, Massachusetts, USA). A bright red color filter (#026 Bright Red) was obtained through Amazon.com and cut into 6 mm disks using a 6 mm punch press. The manufacturer of the red color filter is unknown, but we measured its absorbance spectrum and found that it matches the one used in LUMOS sensor [7]. The 6 mm diameter optics were rolled into one package using black masking tape and inserted into the optics holder. The 5 mm square filter was glued in place using a piece of 9474LE 300LSE adhesive transfer tape (3M, Maplewood, Minnesota, USA). Surfaces that could either leak light or reflect signal were painted by acrylic paint containing PBk7 carbon black pigment (Utrecht Artists Fluid Acrylic Paint, Carbon Black, from Utrecht Art Supplies, New York City, New York, USA).

The Pd-TFPP dye was deposited on a sandblasted and silanized glass plate (10mm Diameter 120 Grit Ground Glass Diffuser by Edmund Optics, Barrington, New Jersey, USA) in a PTFE AF 1600 (Sigma-Aldrich, St. Louis, Missouri, USA) matrix (see the S1 Protocol for a detailed description of the procedure). Silanization renders the glass substrate hydrophobic and allows better bonding of the PTFE matrix containing the luminescent dye. The glass plate was fixed in a 3D printed rhodium plated brass holder manufactured by ShapeWays (New York City, New York, USA) alongside with four gold-plated NeFeB disk magnets (D040A-AU from Amazing Magnets, Anaheim, California, USA). The magnets enable the secure attachment and easy removal of the sensor patches from the experimental vessels. Thus, when experiments require sterility, the experimental vessels can be autoclaved, and the sensor patches can be mounted on the inside of the autoclaved containers. The sensor patches themselves can be sterilized by an overnight incubation in an 70% ethanol solution.

### Analog front end

**Fig 5:**
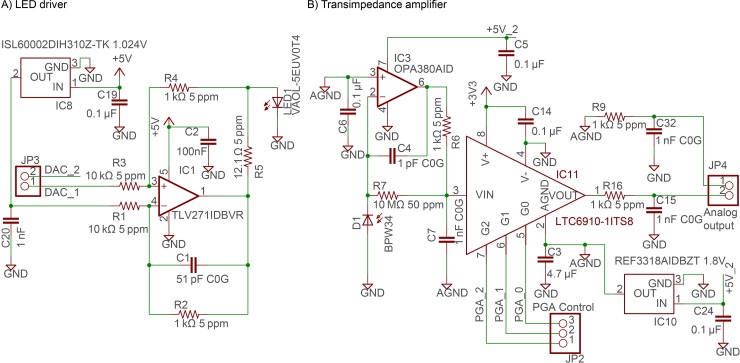
Electrical schematics of the main components of the analog board. A) LED driver circuit. B) Transimpedance amplifier with a programmable gain amplifier. Full schematics are available in the S1 File. Temperature coefficients (in ppm) and dielectric specifications for critical resistors and capacitors are listed in the S1 File as well.

The central part of the sensor system is the analog front-end board that handles signal modulation, reception, amplification, and filtering. The system follows the general layout from Fig 2 and the electrical schematic is shown in Fig 5.

The modulation is performed by voltage-to-current converters based on TLV271 (Texas Instruments, Dallas, Texas, USA) operational amplifiers. The output range of the DAC on the Arduino DUE board is 0.55–2.65 V. Of this voltage, 1.024–2.65 V (due to the use of a 1.024 V reference voltage for subtraction) is used to produce 0–13.5 mA of LED drive current. The LED and the luminescent dye age faster when the utilized power is higher, so it is advisable to keep the operating current as low as possible. We found that 20% of the operational range, corresponding to the peak current of 2.7 mA and average current 1.35 mA, was sufficient to reach the performance levels described in this article.

The readout schematic was built around an OPA380 transimpedance amplifier (Texas Instruments, Dallas, Texas, USA). The amplifier was configured as a 2-pole Butterworth filter according to the manufacturer’s datasheet [17]. The cutoff frequency was kept as high as possible (50.3 kHz) to minimize the phase shift while maintaining amplifier stability. The minimal stable feedback capacitor was found to be 1 pF when the gain was set by a 10 MΩ resistor. The ADC of the Arduino Due is sampled at 342 kHz, allowing 2048 samples per period for a 167 Hz signal. This oversampling of signal reduces the noise in phase measurements in comparison to the 64 samples per period used by the LUMOS sensor, which also used 167 Hz modulation [7].

Because the signal intensity can change depending on environmental conditions, a programmable gain amplifier (PGA) is required to increase the dynamic range of the microcontroller ADCs. We used LTC6910-1 (Linear Technology, Milpitas, California, USA) that allows up to 100x signal amplification in 7 pin-selectable steps. The system’s frequency response changes with the amplification settings, so the gain was kept constant during a single measurement.

To maximize the stability of the system and minimize leakage currents, we encircled the inverting input of the transimpedance amplifier and all sensitive components on both sides of the PCB a driven guard plane that was connected to the non-inverting input of the amplifier. We also used a piece of copper metal foil tape to connect the aluminum tube housing the optical components to the guard plane. Without this the sensor noise can increase by a factor of 10 or more in noisy environments. The guard plane is driven at 1.8 V by a low-noise low dropout linear regulator that also acts as the bias voltage of the photodiode. To minimize power supply noise coupling, the exciting LED drivers and the receiving electronics are powered by separate buck-boost regulators. To minimize any effects due to ground resistance and absorbed noise, we used ground connection vias to separate the sensitive parts of the electronics board. All capacitors in the transimpedance amplifier and signal filtering use C0G (NP0) dielectrics to minimize the effects of temperature dependencies and aging. X5R or X7R dielectrics were used in power decoupling capacitors. In addition, all sensitive resistors were selected to have as small temperature coefficients as possible. The use of low drift components reduced the effect of temperature-induced fluctuations approximately 2.5 times.

Printed circuit boards (PCBs) were designed in AutoDesk E.A.G.L.E. and manufactured by OSH Park (Lake Oswego, Oregon, USA) using FR4 insulators. All the circuit boards have 2 layers to simplify production and debugging. The schematics and board files are attached in the S1 File.

### Other components and mechanical assembly

The mechanical structure of the sensor is critical for its usefulness. We developed attachment strategies to allow the use of the system with a large set of experimental containers, while maintaining reproducible alignment conditions to reduce sensor drift. Our applications used commercially available bottles, so we designed attachments for these. This allowed the sensor to be integrated into standard laboratory procedures.

**Fig 6:**
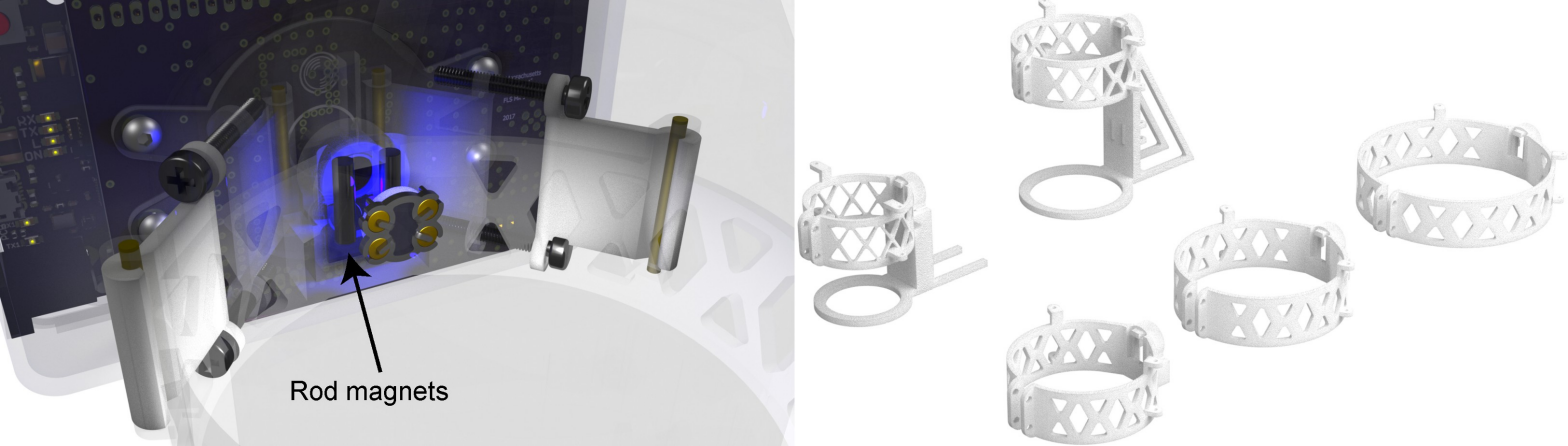
Attachment system. (Left) Close-up rendering of the attachment system for a 250 mL medium bottle. Two vertical rod magnets are used to align the sensor patch. Plastic parts are rendered as partially transparent. (Right) The full set of designed attachments.

The mechanical assembly is easily and economically manufacturable using 3D printing with the selective laser sintering of nylon as the preferred method. The optics assembly (in the S3 File) contains two 2-mm diameter brass rod as hinges used to attach customizable clamps that can be clamped to different bottle and container attachments. A close-up of an attachment setup can be seen in Fig 6. The S4 File contains the designs for attachments for 60 mL serum bottles, 150 mL serum bottles, 50 mL media bottles, 250 mL media bottles and 500 mL media bottles. All these models contain bar magnets (R500A-DM from from Amazing Magnets, Anaheim, California, USA) to allow reproducible alignment of the sensor patches.

### Software

**Fig 7:**
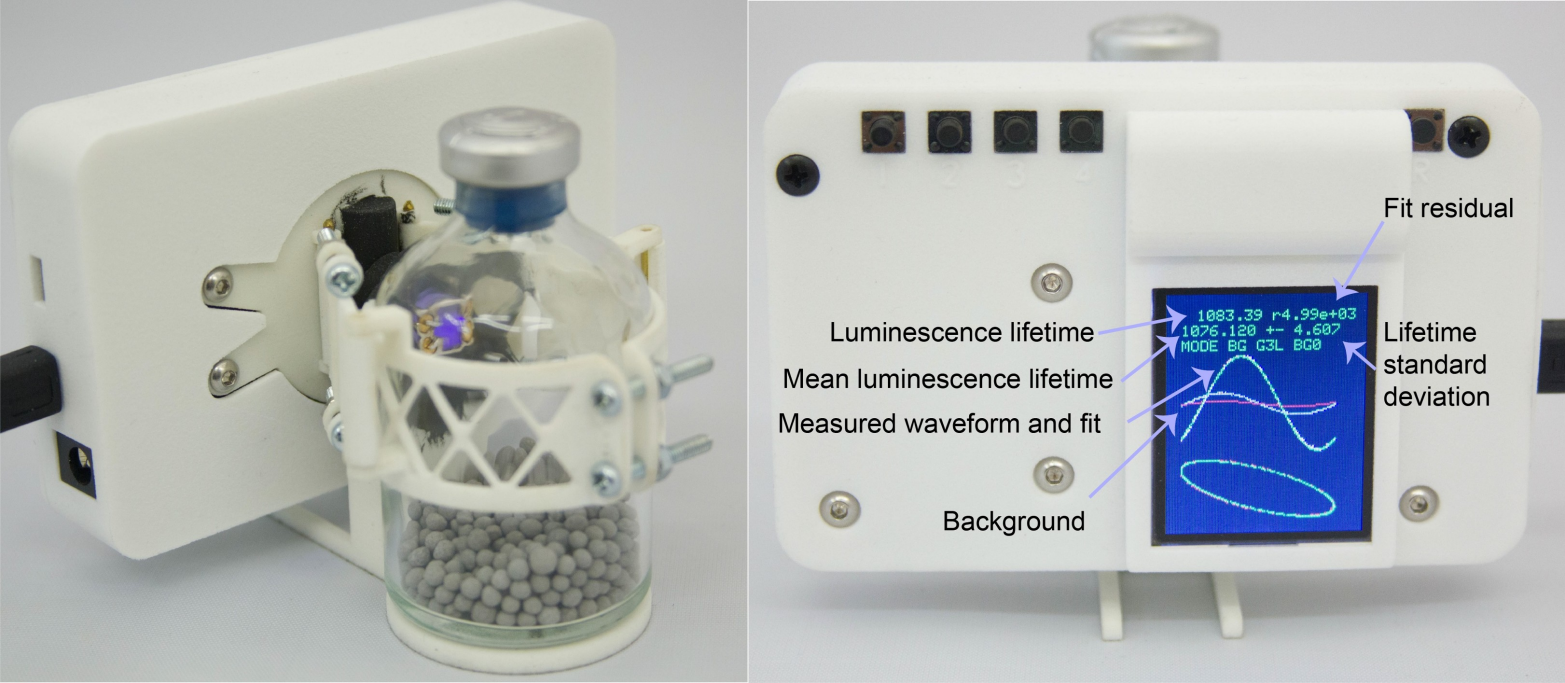
Finished sensor system design attached to a serum bottle. Right part shows the screen and highlights the most important information shown. A highly degraded sensor patch was used to make the background clearly visible. The background is negligible compared to the luminescence for a non-degraded sensor patch.

The documented software for the Arduino Due is provided in S2 File. The software handles LED modulation and signal acquisition. An LCD screen (1.8 inch TFT from SainSmart, sainsmart.com) and buttons provide a simple user interface (see Fig 7).

The signal processing part of the software collects the data and performs the single-frequency Fourier transform. During signal acquisition, all unnecessary modules on the SAM3X8E microcontroller and the ESP8266 module are disabled to minimize interference with the readout electronics. The microcontroller interrupts are also disabled and all operations take a constant number of clock cycles to maintain a uniform timing of the signal generation and acquisition. To maximize the readout speed, direct registry access is used instead of Arduino standard library functions.

The software handles the storage of the background signals including the photodiode signals obtained when the sensor patch is exposed to room air and gain control. Although automatic gain control can be used, a constant gain is recommended because the change in gain can cause a change in phase shift, in addition to invalidating the measured background, and therefore in the measured luminescence lifetime can change. Configuration data and background signals are stored in the SD card attached to the LCD screen module, because Arduino Due lacks built-in nonvolatile storage.

The measured data points are transmitted from the sensor to a computer either over an emulated serial port (physical USB connection) or over Wi-Fi with UDP data packets. The use of a Wi-Fi connection enables the sensors to be situated independently from the logging computer, for example in anaerobic chambers or incubators. UDP packet transport was also found to be more reliable during long duration experiments than USB data transfer, as Arduino Due occasionally loses the ability to connect over serial port and requires manual removal and re-insertion.

The user interface allows the users to capture, store, and erase background signals, set the gain of the PGA, and activate the reference LED. The screen also displays the last captured waveform and associated data, such as the measured luminescence lifetime and noise characteristics, for simplified set-up of experiments. A picture of the user interface is shown on Fig 7. Still, we found wireless or wired data transmission and logging to be the preferred methods for data acquisition.

## Calibration

Accurate calibration is critical for measuring the partial pressures of oxygen lower than 100 μbar. We developed the following protocol:

1. The experimental vessel is flushed with O_2_-scrubbed N_2_ (Nitrogen high purity 300 from Airgas, Hingham, Massachusetts, USA, containing less than 5 ppm of oxygen, which was passed through a Model 1000 Oxygen Purifier from Chromatography Research Supplies, Louisville, Kentucky, USA to reduce oxygen content) at a high flow rate.
2. The flushed vessel is sealed for 12–24 hours to allow the inflow of oxygen by diffusion to stabilize.
3. Immediately before the calibration, the vessel is flushed with oxygen-free N_2_ again.
4. A known volume of water that was bubbled with humidified compressed air as [18] is injected into the experimental vessel using a syringe. Additional volumes of the air-saturated water are added at measured time intervals to establish a set of calibration points.
5. The vessel is flushed with a reference gas that contains a known mixing ratio of oxygen at a known final pressure. We used 75 ppm O_2_ in N_2_ reference gas (Airgas, Cheshire, Connecticut, USA) and equilibrated the system with the atmospheric pressure. To ensure that the equilibrated partial pressure of 76 μbar is attained in the bottle, the bottle must be repeatedly flushed and equilibrated until the final concentration converges. For high accuracy measurements, the ambient atmospheric pressure should be measured and compensated for.

**Fig 8:**
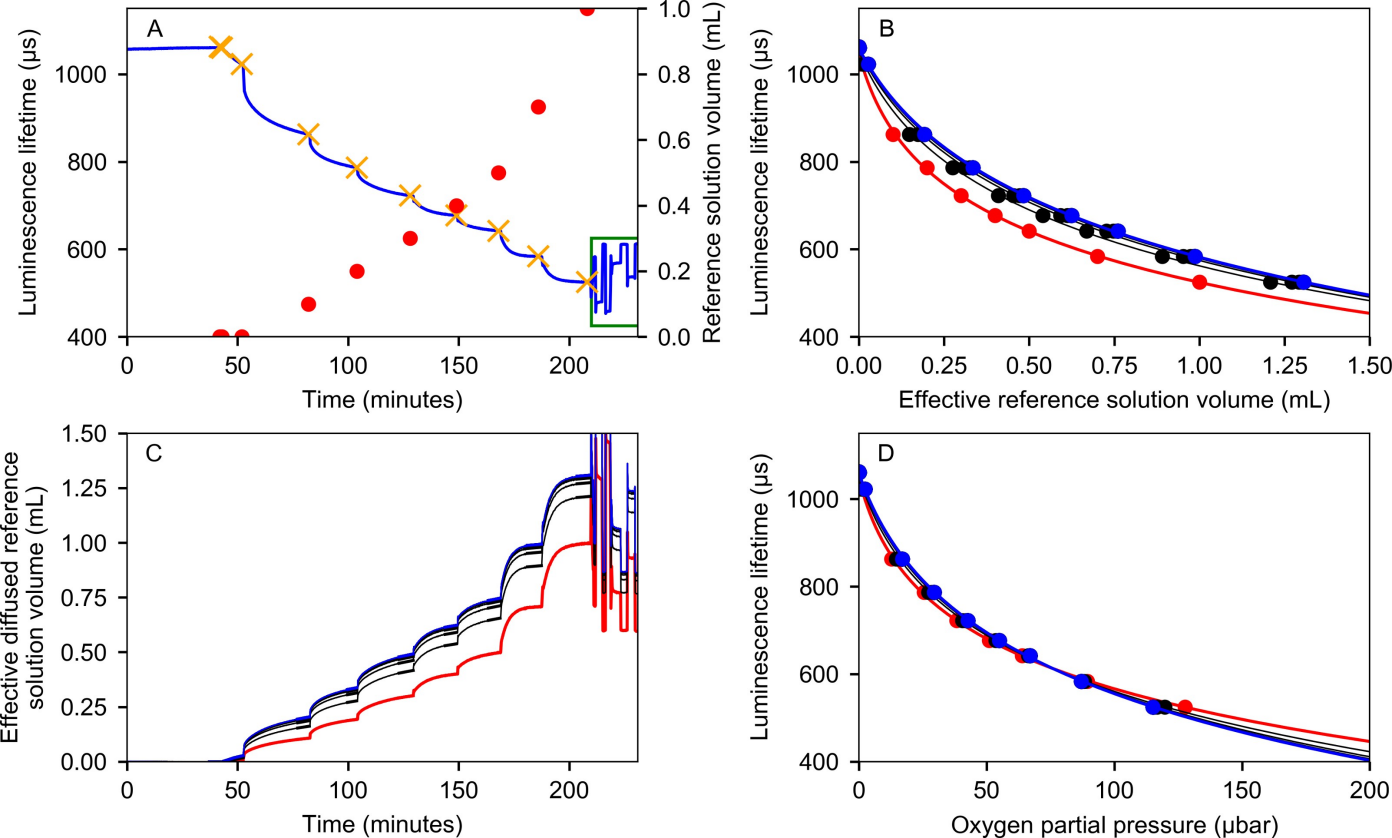
A graph illustrating the calibration procedure. (A) Behavior of luminescence lifetime during calibration (blue). The total volume of liquid injected is shown as red dots, orange crosses indicate injection points on the lifetime graph. After injections, the bottle was flushed with 75 ppm oxygen reference gas and equilibrated with the ambient pressure (green rectangle). (B) The luminescence lifetime of the dye as a function of the effective injected reference liquid volume. Red line is the initial approximation and blue line is the final approximation that compensates for the unintentional inflow of oxygen. (C) Effective amount of oxygen in the gas phase in the units of the injected volume of the reference solution. Red line shows the initial approximation and blue the final one. (D) Calibration curves for the luminescence lifetime as a function of the partial pressure of oxygen with 75 ppm oxygen reference gas in N_2_ used a calibration point. Red is the initial approximation and blue the final one.

The data obtained by steps 1)-5) can then be used to find the dependency between the luminescence lifetime and the partial pressure of oxygen inside the bottle. Fig 8 shows the behavior of the sensor during the calibration that involved the injection of 1 mL of oxygen-saturated water into a 60 mL serum bottle in five 0.1 mL steps, a 0.2 mL step and a 0.3 mL step. The dependency of the luminescence lifetime on the concentration of oxygen is smaller as the partial pressure of oxygen in the bottle increases. Therefore, the injection of larger volumes at later time points reduced the calibration time. The partial pressure of oxygen inside the bottle is larger than expected due to the water injections alone because additional oxygen diffuses through the rubber stopper over the course of the experiment.

To obtain a correct calibration curve and compensate for this effect, we developed an iterative calibration algorithm:

1. A rough calibration is obtained by neglecting unintentional oxygen inflow. To obtain this calibration, the luminescence lifetime is measured before each water injection and related to the volume of water injected until that point. The final lifetime value is recorded after the last injected volume has equilibrated with the headspace, resulting in the red line on Fig 8B. The amount of the injected water is then converted into the expected oxygen concentration in the bottle headspace by multiplying it with the partial pressure of oxygen in the reference gas divided by the amount of water that would need to be injected in order to get the corresponding luminescence lifetime. The result of this calibration can be seen as the red line on Fig 8D. The luminescence lifetime in the presence of 75 ppm oxygen reference gas was 612.4 microseconds.
2. The preliminary calibration (Fig 8B) is used to estimate the effective amount of reference liquid in the bottle during the entire duration of the calibration (the blue line on Fig 8A is converted into the red line on Fig 8C). This amount is the sum of oxygen added by the injections of known volumes of water equilibrated with room air and oxygen that diffused through the butyl rubber stopper.
3. The rate of unintentional oxygen inflow is estimated by performing linear regression on the preliminary graph at the injection points (Fig 8C).
4. The estimated rates of oxygen inflow are integrated over time and the calculated oxygen inflow due to diffusion is added to the injected volume of reference solution on Fig 8B. This produces a calibration graph that accounts for the diffusion of oxygen into the system.
5. Steps 3–5 are iterated at least 5 times until the values converge. The intermediate results are shown as thin black lines on Fig 8B, 8C and 8D. The final result is shown as the thick blue line.

The regression function form used for the data shown in the sample calibration was

$$p_{O_{2}}=c_{1}\exp\left[\left(c_{2}-\tau \right)c_{3}\right]+c_{4}.$$ (5)

**Fig 9:**
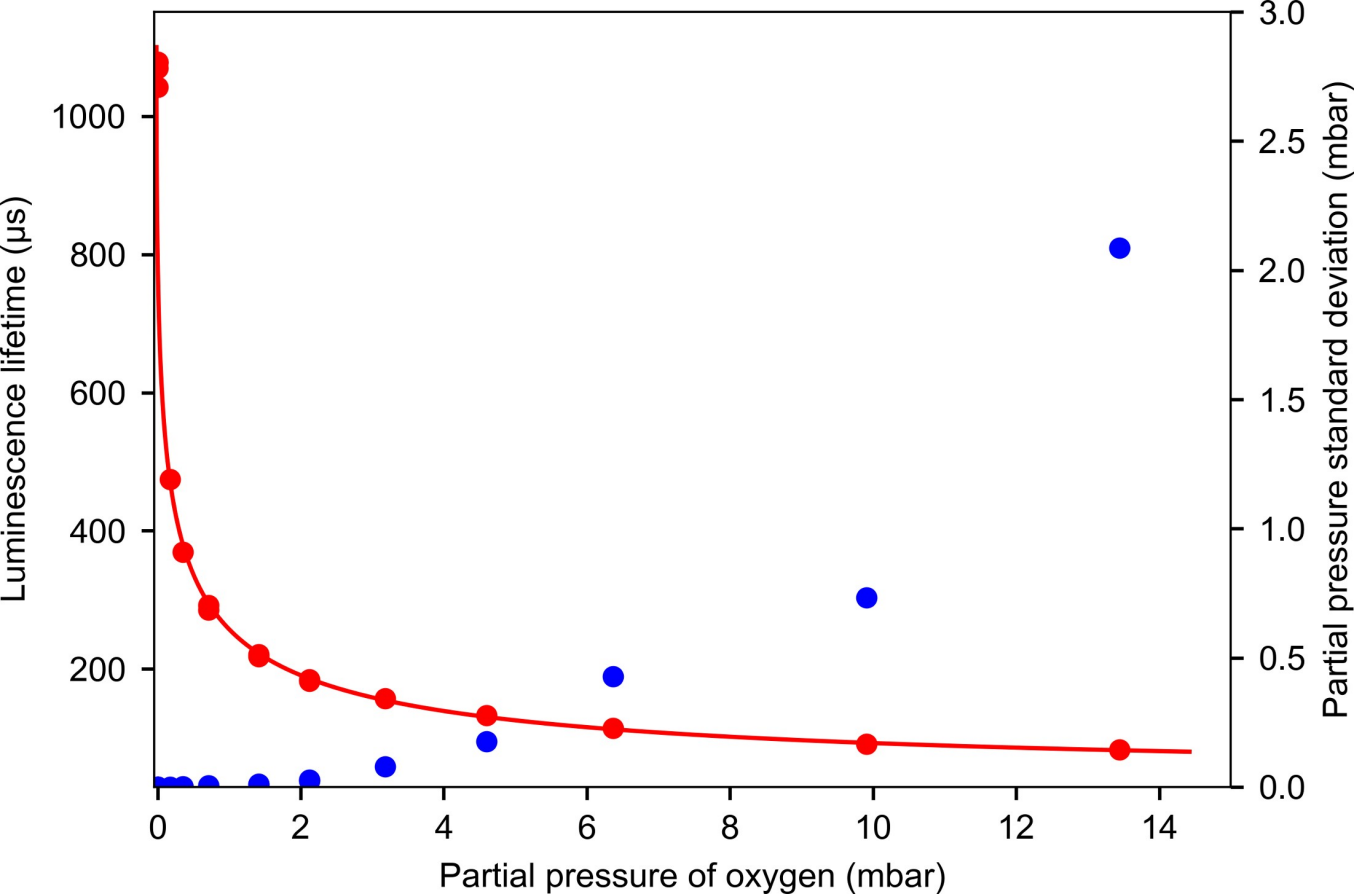
Calibration data at higher oxygen concentrations. Red circles show calibration points, with two independent calibrations overlaid to test repeatability. The solid red line shows the regression function. Blue circles are the standard deviation of measurements and show the sensor noise.

The sensor can measure the partial pressures of oxygen up to approximately 10 millibars, but these measurements require more caution because the effects of all noise and drift sources become amplified due to the weakened signal. Fig 9 shows calibration data from 0–13 mbar oxygen partial pressure range. When equilibrated with water at 25°C, this range is equivalent to dissolved oxygen concentrations in water between 0 and 1.7 μM. The reflected signal passing through the filters limits the upper end of measurable concentrations, because the fluctuations in the intensity of this signal cause noise and drift despite the background subtraction procedure.

For measurements in the upper range, the set of optical filters can be optimized to increase the attenuation of low wavelengths. Different dyes can also be used in this system, such as Pt-TFPP (reported in [7]). At oxygen concentrations higher than 0.1% at ambient pressures, the calibration procedure is much more straightforward because the diffusion of oxygen into the bottle can be neglected: known volumes of room air can be injected into a calibration vessel and the partial pressure of oxygen can be calculated from the total added volume. This calibration can be performed in 30 minutes or less. This is much faster than the calibration in the 0–100 μbar range, which typically takes 2–3 hours in addition to the time required for the inflow of oxygen to slow down, which is typically 12 hours.

The regression function used for the high-range data was:

$$p_{O_{2}}=\frac{c_{1}}{c_{2}-\tau }+\frac{c_{3}}{c_{4}-\tau }+c_{5}.$$ (6)

## Final sensor and examples of applications

**Fig 10:**
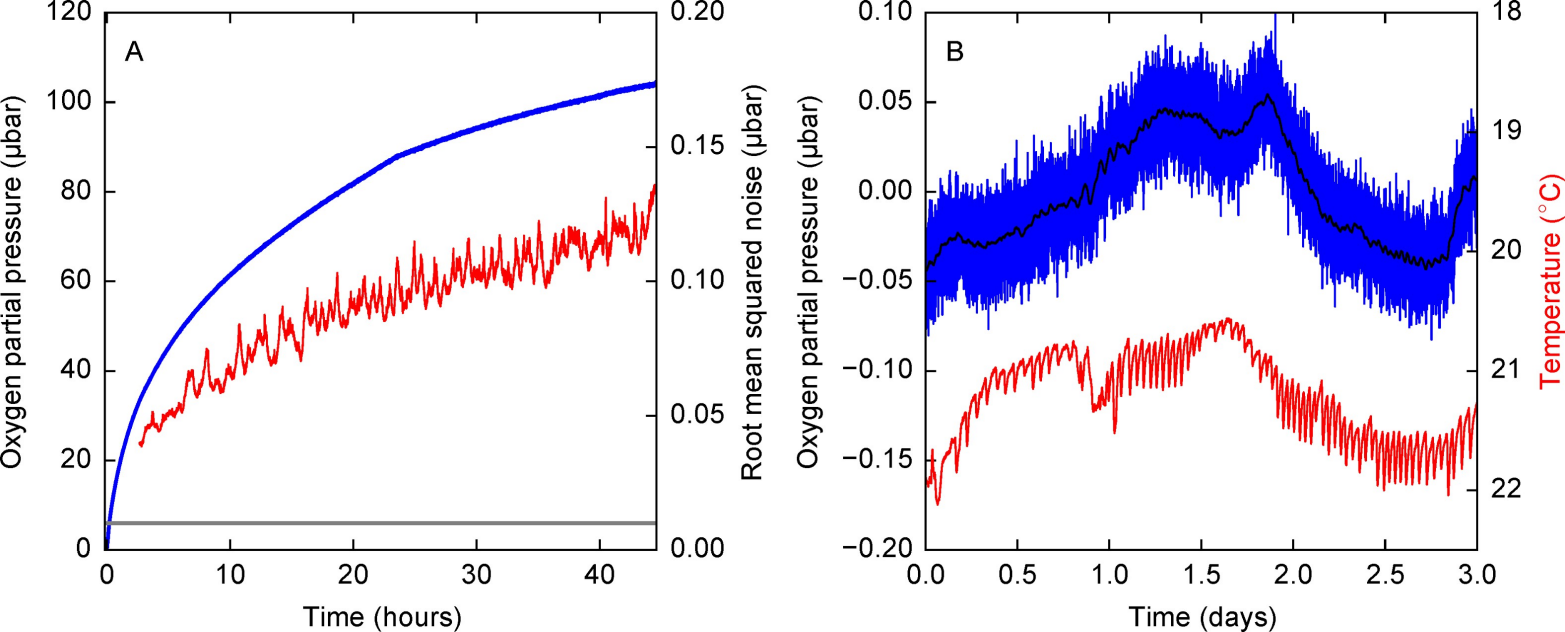
Performance of the oxygen sensor during the measurement of oxygen diffusion into a serum bottle. (A) Blue line shows the concentration readings and red line shows the standard deviation of noise. Gray line shows the standard deviation of noise at zero oxygen concentration in a thermally stable environment (0.01 **μ**bar on average). (B) Stability of partial pressure measurements, when the partial pressure of oxygen is nominally zero in the presence of hydrogen gas and a palladium catalyst in normal laboratory conditions. Blue line shows the obtained calibrated oxygen partial pressure values, the red line shows the temperature in the serum bottle.

To evaluate the noise performance of the assembled sensor, we measured the inflow of oxygen into the same serum bottle that was used to calibrate the system. The root mean squared noise was evaluated by subtracting low pass filtered signal from the original datapoints, i.e., the unprocessed single readings logged straight from the sensor. The noise in the readings of the luminescence lifetime, evaluated as standard deviation, increases with the concentration of oxygen. The noise in the readings varied from 0.01 μbar (root mean squared) when the concentration of oxygen was close to zero to 0.1 μbar (root mean squared) when the partial pressure of oxygen was 100 μbar. The results of the noise quantification experiment are shown in Fig 10A. This leads to a 95% detectability limit of 0.02 μbar.

The lifetime corresponding to zero oxygen partial pressure drifts over the duration of the experiments. We hypothesized that the main causes for this drift are temperature changes and the aging of both the excitation LED and the sensor patch. To test this effect, we used palladium-catalyzed oxygen-hydrogen reaction to keep oxygen concentration at zero. For this, we placed 0.5%wt palladium loaded alumina spheres (Sigma-Aldrich, St. Louis, Missouri, USA) into a butyl-rubber capped serum bottle and flushed the headspace with a gas mixture with 5% hydrogen, 5% CO_2_, and 90% nitrogen (Airgas, Cheshire, Connecticut, USA, this mixture was chosen because it was readily available in the lab for anaerobic chamber operations). Fig 10B shows the zero point drift and the readings from a BMP180 temperature sensor (Bosch Sensortec. Kusterdingen, Germany), inserted into the serum bottle. Over short timescales, the drift follows the temperature changes recorded in the system. The sensor drift corresponding to the changes seen in Fig 10B is within 0.1 μbar over 3 days. This allows us to give a more conservative detection limit estimate of 0.2 μbar for longer period experiments where temperature-induced drift has to considered. This drift can potentially be reduced by thermal compensation, as the main factor behind these fluctuations appears to be temperature. Table 1 shows how the sensor compares to pre-existing ones. The oxygen concentrations in equilibrated water are used, because it is the most commonly reported quantity. The noise values in the table are calculated at 95% confidence level to be comparable to previously published data.

**Table 1:** Comparison between oxygen sensors talked about in this article.

Sensor	Method	Max resolution	Detectability
**Oxygen Sensor Spot [4]**	Optical	160 nM	310 nM
**Trace Oxygen Sensor Spot [4]**	Optical	31 nM	63 nM
**FOSPOR [5]**	Optical	2.6 nM	5.2 nM
**STOX [1]**	Electrochemical		1.6 nM
**LUMOS [7]**	Optical		0.5 nM*
**This paper**	Optical	0.013 nM	0.026 nM

*Level of confidence for LUMOS detection limit is not known.

**Fig 11:**
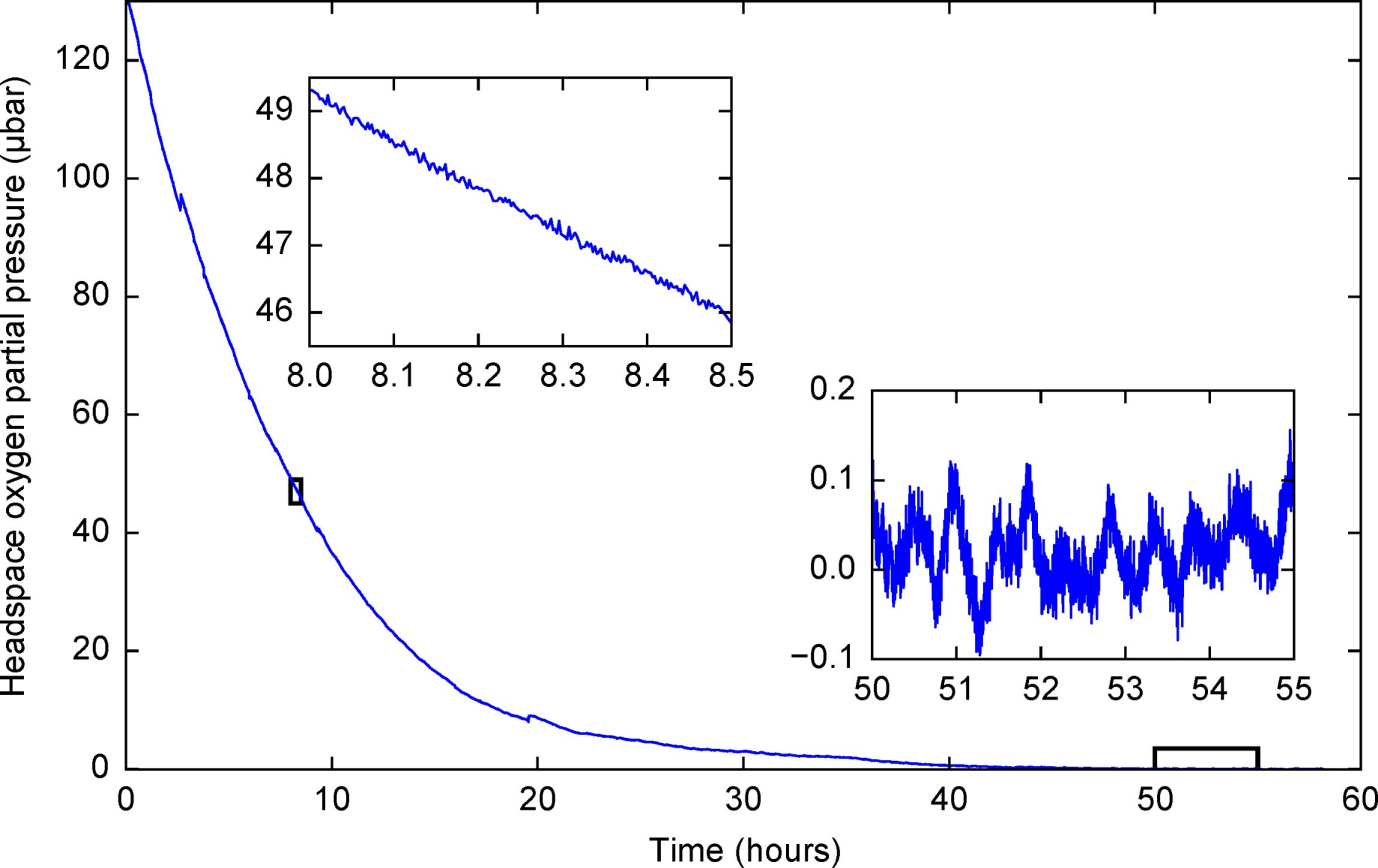
Raw data acquired during the growth of *E*. *coli*. The data were not filtered or smoothed. The two insets show the behavior of the sensor at intervals outlined by the two rectangles and share the same units on the x- and y-axes. The zero point was re-calibrated by flushing the chamber with oxygen-free nitrogen after the end of the experiment.

To demonstrate a second potential application, we monitored the concentration of oxygen in a growing culture of *Escherichia coli* (K12 strain) at low oxygen partial pressures over 60 h, growing in Luria Bretani broth. *E*. *coli* are facultative anaerobes that can reduce the concentration of oxygen in their environment directly, by aerobic respiration, or indirectly, during fermentation (by producing reducing metabolites). The culture bottle used in this experiment had a built-in thermostat to keep the temperature inside the bottle at 37°C. Fig 11 shows the behavior of the sensors and the measured oxygen concentrations. This experiment shows the operation of the sensor when the water droplets condense on top of the sensor patch and when the temperature fluctuates inside the thermostat. The effects of the temperature fluctuations can be seen in the inset that magnifies the time interval between 50 and 55 hours. Effects of water droplets can be seen as the short time discontinuities in the spectrum, the most prominent ones visible at 2.5 and 19.5 hours.

## Conclusion

In this work, we present a design of an open-source trace oxygen sensor for *in situ* measurements in laboratory glassware. The sensor can reliably measure oxygen partial pressures at a low noise up to around 1000 μbar, with the optimum measurement range of 0–100 μbar and the noise standard deviation varying from 0.01 to 0.1 μbar. The noise level can be reduced by increasing excitation intensity at the expense of the LED lifetime and long-term stability. The sensor drift is also very small, corresponding to 0.1 μbar over the course of 3 days. The detection limit for short time measurements is 0.02 μbar and for longer time measurements 0.2 μbar at 95% level of confidence.

The sensor improves upon pre-existing designs in several ways. Not only does the sensor system exhibit a lower noise and detection limit, but it also allows the use of pre-existing laboratory glassware through different attachments. This makes its use practical in microbiology experiments, where standardized, autoclavable containers have to be used. The versatility of the system is further improved by the wireless data logging capability that allows the autonomous monitoring of experiments for months in conditions where direct access to a logging computer is inconvenient, such as anaerobic chambers. We also developed a calibration procedure to fully utilize the measuring capabilities of the system in 0–100 μbar region.

To make this device available for all laboratories, we publish all the resources required to manufacture these sensors in the supplementary information of this article (S1 Protocol, S1 File, S2 File, S3 File, and S4 File). Any updates to the sensor design will be published at http://bosaklab.scripts.mit.edu/trace-oxygen-sensor/. The sensor can be manufactured with minimal instrumentation and the parts that have to be manufactured externally can be fabricated by many independent suppliers.

All in all, the system developed introduces many improvements over the pre-existing systems. The availability of the designs also enables the sensor to be easily adapted to measure luminescence lifetimes of other dye molecules. These adaptations require a simple replacement of the filters in the optics assembly and changes in the modulation frequency.

## Supporting information

S1 Protocol Sensor patch preparation procedure. Contains the procedure for in-house fabrication of sensor patches.
(PDF)

S1 File Design files for electronics. The EAGLE schematics files. (ZIP) For latest version, see http://bosaklab.scripts.mit.edu/trace-oxygen-sensor/.
(ZIP)

S2 File Arduino code for sensor operation. (ZIP) For latest version, see http://bosaklab.scripts.mit.edu/trace-oxygen-sensor/.
(ZIP)

S3 File Design files for mechanical components in STL format. (ZIP) For latest version, see http://bosaklab.scripts.mit.edu/trace-oxygen-sensor/.
(ZIP)

S4 File Design files for bottle holders in STL format. (ZIP) For latest version, see http://bosaklab.scripts.mit.edu/trace-oxygen-sensor/.
(ZIP)
