# Supplementary material for: Open-source sensor for measuring oxygen partial pressures below 100 microbars
Source: PLoS One. 2018 Nov 14;13(11):e0206678. doi: 10.1371/journal.pone.0206678 (PMC6235348; doi:10.1371/journal.pone.0206678)
Supplement: S1 Protocol — Contains the procedure for in-house fabrication of sensor patches. (PDF) [file pone.0206678.s001.pdf]

S1 Sensor patch preparation procedure

The Pd-TFPP dye in this work was embedded into a Teflon AF 1600 matrix. This is similar to the procedure used in (1), but he procedure was custom-developed to use more available components. Teflon AF 1600 is hydrophobic and glass substrates are originally hydrophilic, so the glass substrates were silanized by:

1. Cleaning them thoroughly using ethanol and deionized water.
2. Shaking in a container with 2.5 M NaOH for 24 hours.
3. Sonicating in a sonic bath in deionized water for 10 minutes.
4. Immersing in 80% ethanol for 5 minutes.
5. Dipping into a container of 2% Aquaphobe CF (from Gelest, Morrisville, Pennsylvania, USA) in isopropyl alcohol for 15 minutes.
6. Cleaning the substrates by dipping into ethanol.
7. Storing in a jar with dessicant.

The silanized sensor patches were assembled into the final configuration with the metal holder and the four magnets (Fig 4 in the main article). To coat the patches with the dye, 160 mg of PTFE AF 1600 (from Sigma-Aldrich, St. Louis, Missouri, USA) was dissolved by shaking for 4 days in octafluorotoluene (from Sigma-Aldrich, St. Louis, Missouri, USA) in a fume hood on an orbital shaker. After this, 0.5 mg of Pd-TFPP (from Sigma-Aldrich, St. Louis, Missouri, USA) was added to the solution.

Six microliters of the dye solution were transferred onto the silanized substrate (to the ground side of the glass pieces), already integrated to the holder, in two 15-microliter layers using 5 microliter capillary pipettes (Microcap 1-000-0050 Microliter Pipets from Drummond Scientific, Broomall, Pennsylvania, U.S.A.). The pipettes were also silanized using the procedure described

for glass substrate. The metal holder of the sensor patch acted as a stencil and held the liquid in place while it dried into a thin film. Between the two 15 μl applications, the dye was allowed to dry in a fume hood for a couple of minutes. The dried sensor patches can be immediately used and have at least a two year-long shelf life in standard laboratory conditions and several months of continuous operational life. We did not quantify the operational lifetime, but the longest continuous test run of the sensor was 4 months at 22°C and the sensor patch remained usable during this entire time. Tests performed at higher temperatures indicate faster degradation.

The method described has also been successfully applied for directly spot-coating laboratory glassware (both silanization and dye-coating). The coated glassware also survived autoclaving. Still, using sensor patches has been more convenient in the laboratory conditions.

References

1. Lehner P, Larndorfer C, Garcia-Robledo E, Larsen M, Borisov SM, Revsbech N-P, et al. LUMOS - a sensitive and reliable optode system for measuring dissolved oxygen in the nanomolar range. D'Auria S, editor. PLOS ONE. 2015 Jun 1;10(6):e0128125.
