# Supplementary figures and images for: Open-source sensor for measuring oxygen partial pressures below 100 microbars
Source: PLoS One. 2018 Nov 14;13(11):e0206678. doi: 10.1371/journal.pone.0206678 (PMC6235348; doi:10.1371/journal.pone.0206678)

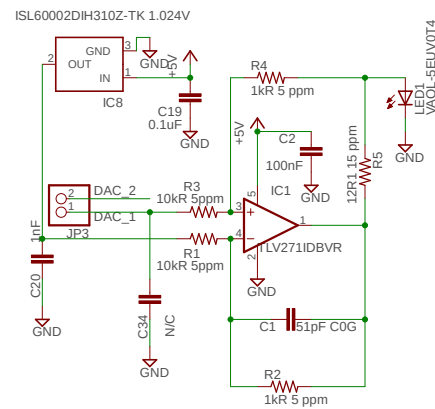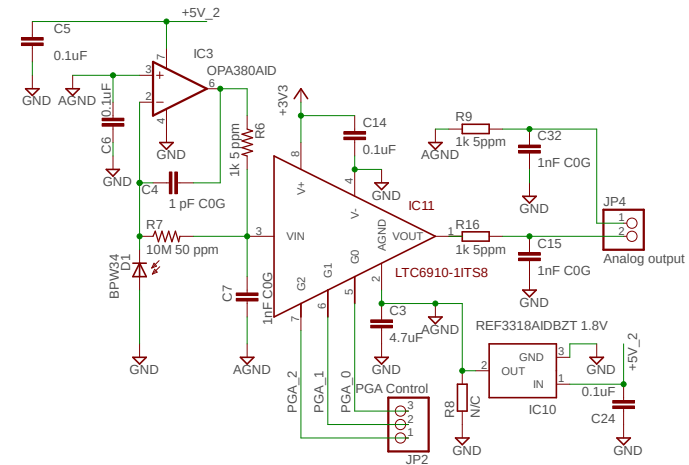

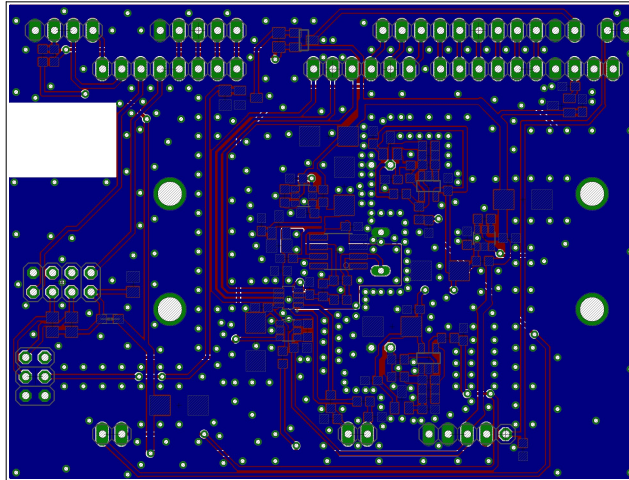

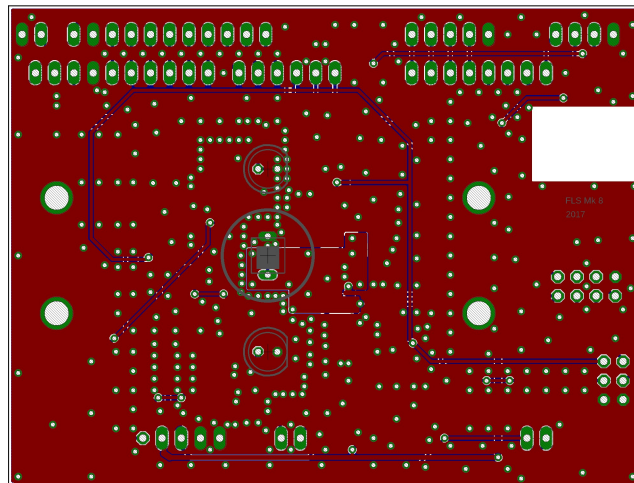

Supplement: S1 File — The EAGLE schematics files. (ZIP) For latest version, see http://bosaklab.scripts.mit.edu/trace-oxygen-sensor/. (ZIP) [file pone.0206678.s002.zip › oxygen_sensor_mk8_final.pdf]
